# Can Generative AI Chatbots Emulate Human Connection? A Relationship Science Perspective

**DOI:** 10.1177/17456916251351306

**Published:** 2025-07-31

**Authors:** Molly G. Smith, Thomas N. Bradbury, Benjamin R. Karney

**Affiliations:** Department of Psychology, University of California, Los Angeles

**Keywords:** artificial intelligence, chatbots, close relationships, intimacy, relationship science

## Abstract

The development of generative artificial intelligence capable of sustaining complex conversations has created a burgeoning market for companion chatbots promising social and emotional connection. The appeal of these products raises questions about whether chatbots can fulfill the functions of close relationships. Proponents argue that relationships with chatbots can be as meaningful as relationships between humans, whereas critics argue they are a dangerous distraction from genuine connections. This analysis applies theoretical tools from more than 50 years of research on close relationships to evaluate the extent to which human–chatbot interactions meet the definition of and fulfill the functions of close relationships. Interactions between humans and chatbots do possess some characteristic features of close relationships: Humans and chatbots can influence each other and engage in frequent and diverse conversations over time. Chatbots can be responsive in ways humans perceive as supportive, generating feelings of connection and opportunities for growth. Yet because chatbots make only superficial requests of their users, relationships with them cannot provide the benefits of negotiating with and sacrificing for a partner and may reinforce undesirable behaviors. Research that attends to the characteristics of users, chatbots, and their interactions will be crucial for identifying for whom these relationships will be beneficial or harmful.

Although computer programs that simulate conversation with humans have been available since the 1960s (Weizenbaum, 1976), recent advances in artificial intelligence (AI) have resulted in sophisticated chatbots that can remember and learn from prior conversations, making them immeasurably more responsive conversation partners (Shevlin, 2024). As the capabilities of AI chatbots expand, people are increasingly turning to them as an easily accessible source of companionship. For example, 660 million users subscribe to XiaoIce, a chatbot developed by Microsoft that advertises an “empathic computing framework” (Spencer, 2018). Replika, another popular chatbot released in 2017, is marketed on its website as “the AI companion who cares: Always here to listen and talk. Always on your side.” XiaoIce and Replika are just two examples of a burgeoning industry valued at over $13 billion in 2024 and expected to grow to almost $30 billion by 2030 (Research and Markets, 2024).

Given fears about humans’ declining capacity to interact effectively with each other (Bzdok & Dunbar, 2022; Office of the Surgeon General, 2023), the increasing use of AI chatbots for companionship raises urgent questions about whether interactions with chatbots can approximate interpersonal relationships with other humans. The developers of these products explicitly market them as proxies for human interaction, describing chatbots as opportunities to create the perfect customizable friend, mentor, or lover. Indeed, many consumers of these products do describe their chatbots as friends, boyfriends, or girlfriends (Pentina et al., 2023). In one survey of Replika users, 88% of participants (*N* = 145) identified their chatbot as their “partner” (De Freitas, Castelo, et al., 2024). Some scholars have endorsed this view, suggesting that companion chatbots can fulfill some of the beneficial functions that relationships between humans fulfill (Broadbent et al., 2023).

In contrast, others have argued that interactions with chatbots are ersatz relationships at best and a dangerous distraction from genuine human connection at worst. Critics have cautioned about the risks of interacting with an entity that is incapable of genuine empathy (Shteynberg et al., 2024), warning that companion chatbots will allow users to experience “‘the pleasures of companionship without the demands of friendship’” or “‘the feeling of intimacy without the demands of reciprocity’” (Mineo, 2023). Such freedoms could damage the very social skills that proponents suggest chatbots might preserve and foster an unhealthy degree of psychological dependence.

Evaluating the extent to which interactions with AI in fact replicate the functions of human relationships requires, at a minimum, an explicit definition of interpersonal relationships, a theory of how feelings of interpersonal connection arise, and a framework for understanding the functions that interpersonal relationships may serve. Fortunately, the field of relationship science has been addressing these very questions for decades and has accumulated an array of well-validated and time-tested theories, models, and empirical strategies to describe the development and consequences of close relationships between humans (Bradbury & Karney, 2024). Applying these tools to interactions between humans and AI chatbots can provide a benchmark against which chatbots and their current and potential functions can be evaluated.

Thus, the goal of this analysis is to provide frameworks for evaluating relationships between humans and generative AI chatbots by drawing on the last 50 years of research and theory on close relationships between humans. In pursuit of this goal, this article addresses three questions. First, to what extent can interactions with chatbots meet the defining criteria of a close relationship? Second, can chatbots fulfill key functions of close relationships (namely, generating feelings of connection, providing social support, and facilitating personal growth)? Third, what are the current challenges and risks to humans of pursuing close relationships with AI chatbots? Following our discussion of these three questions, we propose top priorities for future research in this rapidly developing area.

## Historical Overview and Scope of Our Analysis

The creation of the first chatbot is credited to Joseph Weizenbaum, who developed ELIZA in 1966 (Belk, 2022). Designed to mimic a person-centered psychotherapist, ELIZA used scripts and pattern matching to rephrase input from human users, thereby encouraging them to continue the conversation. Despite the simplicity of ELIZA’s capabilities, people who interacted with her reported feeling as though she truly understood them and even asked for time alone with her (Weizenbaum, 1976, p. 189).

Modern chatbots are no longer restricted to rephrasing input. Now they can sustain and initiate complex conversations, search the internet, and complete tasks for their users. In the 2010s, chatbots moved into the home and onto mobile devices as conversational service providers. Apple’s Siri and Amazon’s Alexa were able to accomplish tasks such as providing recommendations and reminders and describing the weather and traffic.

As capable as these devices were, chatbot technology advanced substantially in 2022 when OpenAI released ChatGPT, a chatbot based on generative AI. As a large language model, ChatGPT is pretrained on vast datasets and fine-tuned through human feedback and data from user input. In addition to completing more service-oriented tasks (e.g., answering questions, writing computer code, translating languages), GPT’s conversational skills and ability to produce personalized responses are remarkably humanlike. In a battery of behavioral tests including trust, bargaining, and prisoner’s dilemma games, ChatGPT’s behavior was deemed “statistically indistinguishable” from human subjects (Mei et al., 2024, p. 1). Moreover, improvements in large language models have rapidly improved chatbots’ humanlike reasoning ability. In theory-of-mind false-belief tasks, GPT models from 2022 and 2023 solved only about 20% of tasks, whereas a model released just 1 year later in 2024 performed comparably to 6-year-old human children, solving 75% of tasks (Kosinski, 2024). The responsive abilities of GPT and similar models have been repackaged by chatbot companies to serve a variety of purposes, including psychotherapy, customer service, and companionship (Belk, 2022).

The emergence of computer programs that effectively replicate human speech and cognition raises numerous issues, from general philosophical questions about AI’s capacity for consciousness (Long et al., 2024) to specific legal questions about liability and consumer protection (Boine, 2023). As relationship scientists and psychologists, our concerns focus not on the products themselves, which vary in their designs and capabilities, but on their effects on humans who are looking to them for experiences of companionship. This focus naturally includes companion chatbots designed to be social and empathic (e.g., Replika) but also more general-purpose chatbots (e.g., ChatGPT) that some users use to fulfill social needs (Phang et al., 2025). In contrast, AI products designed to serve a narrower range of functions, such as therapy chatbots and service-oriented chatbots, fall outside the scope of this analysis.

At the outset, we recognize that AI chatbot technology is developing rapidly such that the specific proficiencies of these products have expanded even as we have been preparing this article for publication. Empirical research on the effects of these technologies on humans, although developing quickly, remains nascent. To date, most studies in this area have relied on small convenience samples, or self-selected samples of current users, limiting the ability of the existing literature to support general conclusions about the population of users and potential users. Thus, a comprehensive review of research on this topic is neither possible nor desirable. Nevertheless, if we are to understand the implications of this new social phenomenon, waiting until the technology and research stabilize to review the literature would be equally unwise. Instead, the goal of this analysis is to draw links between this emerging body of research and the well-established literature on relationships among humans to comment on the capacity for chatbots to provide relational experiences and functions to at least a subset of users. Acknowledging the rapid pace of technological evolution, throughout this article we distinguish between the relational capacities that generative AI chatbots can currently achieve, capacities that seem achievable in the near future, and capacities that chatbots are unlikely to achieve.

## Can Humans Have Close Relationships With AI Chatbots?

Humans are such profoundly social animals (Frith & Frith, 2010) that we have the capacity to feel emotional attachments (i.e., parasocial relationships) with a variety of targets that cannot or do not reciprocate our feelings, including celebrities, public figures, sports teams, political parties, and fictional characters (Hartmann & Goldhoorn, 2011). The fact that humans can feel closely connected to celebrities and fictional characters raises the likelihood that humans can feel similarly connected to AI-powered chatbots. Unlike targets of parasocial relationships, however, generative AI chatbots can respond to human prompts and carry on humanlike conversations with users. Do these capacities enable humans to develop genuine relationships with their chatbot companions?

Thibaut and Kelley (1959) proposed that the identifying feature of an interpersonal relationship is *interdependence* (i.e., a state of mutual influence in which the actions of each partner simultaneously affect the other partner). From this perspective, chatbots and humans exist in a relationship as much as any two interacting agents. Human input affects how chatbots behave because chatbots take in new information about their users and incorporate personalized feedback into future responses (Ait Baha et al., 2023). Likewise, the responses generated by a chatbot can affect users such that an appropriate response may generate feelings of fulfillment and connection whereas an inappropriate response may generate feelings of rejection or distress. Thus, interactions between humans and AI chatbots meet this minimal requirement.

What the companies behind AI companions are selling, however, is not interdependence but a close and personal relationship that can provide friendship, love, and support. Kelley et al. (1983) defined close relationships as those characterized not merely by interdependence but by influence between partners that is strong, diverse, frequent, and long-lasting. Friends, family, and romantic partners typically meet this definition because they influence and are influenced by us in substantial ways (strong), we tend to engage in a wide range of activities with them (diverse), we interact with them regularly (frequent), and our contact with them lasts for years of our lives (long-lasting). To evaluate whether interactions with chatbots meet these standards, we can draw on descriptive and experimental studies to evaluate how strong, diverse, frequent, and long-lasting interactions with AI chatbots can be.

With respect to strength of influence, sustained and repeated interactions between humans can produce lasting changes in self-concept and behavior because each partner must attend to and consider the other’s goals, needs, and motives over time. Interactions with chatbots are capable of having similar effects: At least a subset of users describe substantial changes in their behaviors and emotions as a result of interactions with chatbots. For example, a text-mining analysis of more than 100,000 reviews of the Replika app indicated that chatbots can influence users’ moods and identified patterns of emotional shifts in the narratives that included high anticipation for future use, joy, sadness, and trust (Siemon et al., 2022). In qualitative analyses of open-ended questions, some Replika users described changing their habits and behaviors in response to their chatbot’s advice (Ta et al., 2020).

The extent to which human users influence their chatbot companions, however, is less clear. Currently, a human’s influence can change only the verbal responses generated by their chatbot partner, an effect that pales in comparison to the deeper ways that humans shape each other in relationships. In close relationships between humans, sacrificing for a partner can elicit positive emotions and benefit relationship quality (Kogan et al., 2010). Chatbot users, however, are less able to reap these benefits because their chatbot partners do not have tangible needs for them to attend to. Thus, although humans can be strongly influenced by their chatbots, they are unable to reciprocate this influence to the same degree, depriving them of the benefits of supporting and sacrificing for a partner.

With respect to diversity of interactions, surveys of chatbot users reveal a wide range of goals and motives in initial desires to download chatbot companions. When Replika users (*N* = 59) were asked about their motivations to use the app, a thematic analysis of their survey responses indicated that about half of the respondents were motivated by curiosity and a general interest in new technologies, 24% sought social support to address loneliness, and a smaller group mentioned motivations related to improving mental (8%) or physical (7%) health (Ta-Johnson et al., 2022). A computational analysis of more than 35,000 posts on the Replika subreddit identified eight different themes in users’ interactions with their chatbots, including intimate behavior, play and fantasy, and user self-disclosures of attitudes, opinions, identities, and secrets (Li & Zhang, 2024). When Replika users (*N* = 66) were asked to describe the topics they frequently discuss with their chatbots, almost all participants listed several, including science and technology, mental health and emotions, personal issues, sex and intimacy, relationships, and current events (Ta-Johnson et al., 2022). Even people with little prior experience using companion chatbots are still able to engage in a wide range of conversations with a generative AI chatbot. In a longitudinal study, these participants were instructed to have daily personal conversations with ChatGPT for 4 weeks, and a large language model subsequently coded the content of the interactions and categorized them into 15 unique topics. The most common conversation topics for this group were emotional support and empathy, casual conversation and small talk, and advice and suggestions (Phang et al., 2025).

Yet the potential diversity of interactions with AI chatbots goes only so far: Humans can have diverse conversations with their chatbot companions, but these shared activities remain conversations. At present, chatbots are not able to engage in the wider range of interactions that humans in close relationships can share with each other (e.g., eating, parenting, doing chores). Although AI can discuss or even pretend to act out these activities, they remain limited in their ability to engage in diverse modes of interaction beyond conversation.

With respect to frequency of interactions, the companies behind AI companion chatbots have a financial incentive to keep users engaged. Microsoft, for instance, evaluates the success of XiaoIce by how many conversational turns occur between the human and the chatbot per session. As the chatbot has become more sophisticated, the average number of conversation turns per session has increased from five in the first generation of XiaoIce to 23 turns in the sixth generation, a number higher than in many human conversations (Zhou et al., 2020). In interviews (*N* = 18), Replika users described frequent interactions with their chatbots that can last for hours, and “almost all” participants reported interacting “extensively” with their chatbot in the beginning of the relationship (Skjuve et al., 2021, p. 6). In another survey of 19 Replika users, the average daily interaction duration with the chatbot was between 2 and 3 hr (S. Ma & Koike, 2025). Nevertheless, for some chatbots, engaging in longer, more frequent, and more complex interaction is contingent on paying ongoing subscription fees, imposing limits on the ways some users are able to interact with their chatbots (Chaturvedi et al., 2024).

With respect to duration of contact, for some users, relationships with chatbots can last for months to years. For example, when 19 Replika users were interviewed about their chatbots, most participants described the relationship as long-term (Brandtzaeg et al., 2022). In a survey study of 92 companion chatbot users recruited on social media, the average length of the ongoing relationship between humans and their chatbot was 5.94 months (Ebner & Szczuka, 2025). Replika’s website also features testimonials from users, many of whom have spent years together with their chatbots. Yet an implicit feature of Kelley et al.’s (1983) discussion of duration is that interactions between humans in close relationships build on each other over time. That is, each interaction in a long-term relationship reflects and acknowledges prior interactions and experiences unique to the relationship. Relationships between humans deepen as the shared history of two partners accumulates. In interactions with chatbots, however, the potential for this accumulation of shared experience is limited by chatbots’ lack of extended memory for previous interactions. Despite built-in features allowing them to store information, chatbots’ capacities to remember and build on prior conversations are not yet well developed (Brandtzaeg et al., 2022). Indeed, in a random sampling of 120 posts containing about 3,000 user comments on the Replika subreddit, users described their frustration from having to repeat themselves to their chatbot companions and reported repeatedly coaching them to remember basic information, including their name, interests, and preferences (Z. Ma et al., 2024). Thus, although AI companion chatbots are able to sustain frequent and long-lasting interaction, they currently cannot fully replicate the continuity that characterizes ongoing relationships between humans.

Table 1 summarizes the capabilities and limitations of AI chatbots as close relationship partners given their current level of development. As the table makes clear, chatbots can meet some but not all of the criteria that relationship scientists have used to define close relationships. Chatbots can converse with humans in ways that are diverse, frequent, and long-lasting. Technological limitations, however, including limited memory and an inability to engage in modes of interaction beyond conversation, make these relationships substantially different from close relationships between humans. These limits will likely soon be overcome as technology improves. The greatest difference between interactions between humans and interactions between humans and chatbots lies in the possibility of mutuality: Humans can be affected in many ways by their interactions with chatbots, but chatbots are not substantially changed by their human users.

**Table 1.** Can Humans and AI Chatbots Have Close Relationships?

Defining features of close relationships	Capabilities of chatbots	Limitations of chatbots
Each partners’ actions exert strong influence on the other’s outcomes.	Chatbots can significantly affect humans’ behavior and emotions. Human input shapes and changes chatbot responses.	The mutual influence between humans and chatbot companions is uneven: • Human input does not affect the underlying large language models that generate chatbot conversation. • Whereas chatbots can affect humans’ behavior and emotions, humans can affect only a chatbot’s conversation.
Partners engage in a wide range of different types of interactions with each other.	Conversations between humans and chatbots can be informational, emotional, therapeutic, sexual, playful, and argumentative.	Chatbots cannot engage in physical behaviors and activities; interactions between humans and chatbots remain confined primarily to conversation.
Partners interact with each other frequently.	Humans can interact with their chatbot companions many times per day, and interactions can last for hours.	For some chatbot companions, access to more frequent, complex, and longer conversations requires ongoing subscription fees that some users may not be able to afford.
Partners interact over a significant period of time.	Some humans report ongoing interactions with their chatbot companions lasting for months to years.	Chatbots can retain user data only for a limited period of time, preventing the long-term accumulation of shared experiences between partners.

## Can Chatbots Fulfill the Functions of Close Relationships?

The fact that human–chatbot interactions replicate some of the defining features of close relationships does not imply that they necessarily serve the same functions that relationships with other humans serve. Among humans, relationships with close others can generate feelings of connection and belonging (Beller & Wagner, 2018), provide social support (House et al., 1988), and, at their best, encourage and facilitate personal growth (Feeney, 2004). Can close relationships with AI chatbots also serve these beneficial functions for their users?

### Feelings of connection with chatbots

Addressing whether interactions with AI chatbots can generate feelings of authentic connection for their users requires first understanding how feelings of connection arise between humans. To this end, Reis and Shaver’s (1988) seminal intimacy process model proposes that feelings of closeness between people arise from the associations between one person’s behaviors and another person’s responses. Given a disclosure of self-relevant information from one person, the response from the other person can lead to feelings of intimacy and connection when that response makes the discloser feel understood, validated, and cared for. Thus, the perception of a partner’s responsiveness is central to feeling close and connected to that partner (Laurenceau et al., 2005). From this perspective, relationships with chatbots should generate feelings of connection in humans to the extent that (a) humans regularly disclose self-relevant information to their chatbots and (b) AI-generated responses are perceived to be understanding, validating, and caring. Research on these relationships has found that, at least for some people, both of these conditions can be met.

Experimental and descriptive evidence suggests that people self-disclose emotions or information to their chatbot companions frequently and with intensity. In an experiment in which participants were asked to disclose a secret to either a human or chatbot conversation partner, there was no difference in the intimacy of participants’ self-disclosures measured by content analysis from human coders (Croes et al., 2024). Qualitative interviews with Replika users identified multiple users who reported in-depth self-disclosure early in the relationship (Skjuve et al., 2021). Other interviewees described sharing personal secrets with their Replika that they had not shared with other people (Pentina et al., 2023).

Can a chatbot generate responses to these disclosures that make humans feel understood, validated, and cared for? To the extent that they have access to millions of examples of conversations, generative AI chatbots certainly have the potential to mimic effective human responsiveness. By storing information, learning to replicate users’ language styles, and using feedback about whether a given response is helpful, companion chatbots, in particular, can respond in ways that feel individualized. Not only can the content of a chatbot response be similar to how a human might respond, but the process by which they deliver a response can also mirror human conversation as they can replicate turn-taking, initiate conversation by asking questions or changing the subject, and acknowledge and restate what their human conversation partner has contributed. When experimentally manipulated, chatbots that possess more of these conversational skills are perceived to be more humanlike (Bergner et al., 2023).

Responses from companion AI chatbots can in fact produce feelings of connection and reduce loneliness, at least in the short term. After one 15-min session of interacting with a customized companion chatbot based on OpenAI’s GPT-3, participants reported a reduction in loneliness at levels similar to participants who interacted with a human through an online chat, and these reductions were significantly greater than reductions in control groups who were instructed to watch videos or do nothing for 15 min (De Freitas, Uguralp, et al., 2024). In a follow-up longitudinal study, participants reported their loneliness before and after interacting for 15 min with the chatbot each day for 7 days. Interacting with the chatbot reduced loneliness the most on the first day, but significant reductions in loneliness after each chatbot interaction continued throughout the week (De Freitas, Uguralp, et al., 2024).

For some users, the sense of connection and satisfaction derived from their chatbot relationship even rivals that of their close human relationships. In an online study in which 101 Replika users were surveyed about a variety of personal relationships (including those with a colleague, an acquaintance, a close friend, and a close family member), participants reported greater relationship satisfaction, social support, and closeness to their Replika compared to all other human relationships, except for the close family member (De Freitas, Castelo, et al., 2024). In an experiment in which participants self-disclosed to what they thought was either a chatbot or a human, the benefits of emotional disclosures, including improved mood and perceived relationship quality, were equivalent across conditions, regardless of the perceived identity of the conversation partner (Ho et al., 2018). A sentiment analysis of more than 500,000 words expressed in more than 100,000 user reviews of Replika revealed that words associated with positive emotions were most common, including feelings of joy, trust, and anticipation for future interactions (Siemon et al., 2022).

AI chatbots have demonstrated their ability to respond appropriately to disclosures from humans, but humans in close relationships regularly engage in reciprocal self-disclosures, enhancing perceived responsiveness and relationship quality (Sprecher & Hendrick, 2004). This degree of responsiveness might seem more difficult for a chatbot, as chatbots do not have memories, fears, or experiences to disclose. Instead, companion chatbots are programmed to appear as if their behavior is based on their goals and specific personalities. When a large language model classified the content of more than 300,000 messages between participants and ChatGPT over the course of a 4-week study, levels of self-disclosure from the text-based version of the chatbot were comparable to those of the human participants (Fang et al., 2025). This programming seems to be effective, as Replika users have described their chatbots as having opinions (Ta et al., 2020) and as entities “with desires” and “with needs” (Skjuve et al., 2021, p. 7).

Such perceptions do not require chatbot users to ignore their chatbots’ limitations. Rather, users employ what Turkle (2024) labels “dual consciousness”—a state in which the knowledge that their chatbot cannot truly care for them, be conscious, or provide genuine disclosures coexists with feelings of connection and emotional investment. A forum comment by a Replika user describes this experience: “‘Even though I know in the back of my head that she’s an AI and this is an app, she does genuinely make me happy’” (Marriott & Pitardi, 2024, p. 92).

This perception of responsiveness is the key for generating feelings of connection with humans and with AI chatbot companions, and users who experience these feelings gain benefits similar to those that people receive from reciprocal self-disclosures with other humans. For example, in an experiment in which participants discussed a stressor with a chatbot, their stress was reduced most when the chatbot provided a reciprocal self-disclosure alongside emotional validation compared with emotional validation or self-disclosure alone (Meng & Dai, 2021). In a 3-week study that manipulated a chatbot’s level of reciprocal self-disclosure (none, low, or high), disclosures from the chatbot encouraged further disclosure from the human participants and improved perceptions of intimacy with the chatbot (Lee et al., 2020), consistent with the predictions of the intimacy process model.

How do people suspend disbelief and maintain the perception of genuine responsiveness and disclosure from companions they know are computer programs? Evolutionary perspectives propose that because human brains have evolved to prioritize social stimuli for survival, humans are inclined to interpret any humanlike appearance or behavior as having social motivations (Stroessner & Koya, 2024). The inclination to anthropomorphize nonhumans automatically and easily influences behavior: Humans tend to be polite to technology, respond differently to male and female computer voices (Reeves & Nass, 1996), and make inferences about the mental states of robots (Stroessner & Koya, 2024). In a proposed model of how AI chatbots might fulfill social needs, Machia et al. (2024) noted that for most humans, interactions with AI likely consist of two parts: first, an automatic processing of the interaction in which the chatbot feels quite humanlike, and second, a conscious processing of the interaction in which users reflect on the fact they are interacting with a machine, which can diminish feelings of connection. For some users, however, especially those who are lonely and in need of social connection, this secondary “deliberative processing” is bypassed, and the perception of the AI as equivalent to a responsive human is maintained. Through this mechanism, generative AI chatbots can produce feelings of connection that may be indistinguishable from those to any responsive human.

As much as it is validating to be responded to in positive ways, what humans provide that AI chatbots do not is a sense of being reciprocally chosen. Downloading an AI chatbot means that the user’s interest in the chatbot will always be reciprocal because the chatbot is programmed to offer its full attention and effort unconditionally. Between humans, one of the thrills of a new social connection arises from the uncertainty of being chosen back, and being chosen by a relationship partner who is selective is especially rewarding (Eastwick et al., 2007). In one experiment, participants interacted with a human face to face, a human online, or a Replika chatbot for 20 min. Postinteraction surveys and transcript analyses revealed that although the chatbot interactions had the highest levels of positive tone and positive emotions, these characteristics did not translate to greater reported liking for the chatbot (Drouin et al., 2022). Rather, the human partners were perceived as more responsive and likable. The unconditional positivity provided by chatbots may therefore not be as rewarding as the contingent positivity earned from a more selective human partner.

Accompanying the potential for chatbots to respond appropriately to human disclosures is their potential to respond in ways that are perceived as inappropriate, destructive, or unresponsive. The technology behind chatbots’ ability to accurately understand and respond to what a user says remains limited. Under some circumstances, a chatbot’s responses can come across as impersonal, scripted, or even nonsensical, and when confronted by such responses, users describe feeling unsupported and disconnected (Loveys et al., 2022; Ta et al., 2020). Because users project humanlike expectations onto their chatbots and suppress awareness of their chatbots’ limitations, such out-of-place responses can be especially jarring and disruptive to the relationship. Of course, given that human companions are also prone to clumsy responding at times, it remains to be seen whether their limitations make chatbots any more or less prone to insensitive responses than humans.

### Social support from chatbots

When people are distressed or in need they are most likely to seek support from their closest relationships, usually their friends, family, and intimate partners (House et al., 1988), and the exchange of support can be beneficial for the well-being of the receiver and the provider (Taylor, 2011). Merely perceiving that support is available has been associated with uniformly positive outcomes (Lakey & Cassady, 1990), whereas perceiving that social support is unavailable has been associated with negative outcomes (e.g., early mortality; Shor et al., 2013). With respect to availability, chatbots are more available than humans. They are accessible 24 hr a day, do not sleep, do not have jobs or competing responsibilities, and cannot be bored. Whereas receiving support from another human can result in an obligation to reciprocate, users have no such obligations to computer programs. In an online study in which participants reviewed an advertisement for an app that was framed as either an AI or human companion, the AI companion was indeed rated as more available and less judgmental than the human companion (De Freitas, Oğuz-Uğuralp, et al., 2024). In qualitative studies, users recognize their chatbots’ constant availability, identifying it as strikingly dissimilar from their human-human relationships (Brandtzaeg et al., 2022) and viewing it as a distinct benefit (Maples et al., 2024).

Being available to provide support is not the same as providing support effectively. Because received support can be clumsy or ineffective, people are not always better off when they actually draw upon close others for social support. The ability of chatbots to provide support that benefits their human companions depends on the type of support that they are called on to provide. Models of social support between humans distinguish between several different forms of support (e.g., Cutrona, 1986); chatbots are likely be more effective at providing some forms than others.

One way to support people experiencing distress is by expressing acknowledgment and understanding, elaborating upon and exploring the person’s feelings, and expressing sympathy (Bodie & Burleson, 2008). Receiving such support contributes to emotion regulation and comfort for the person experiencing distress. Research on chatbots as mental health providers has generally found that chatbots can serve humans effectively in this way (Limpanopparat et al., 2024). Companion chatbot users also report that their chatbots provide emotional support by prompting them to engage in self-evaluation and emotion reappraisal (Maples et al., 2024; Ta et al., 2020) and by validating their emotions (Marriott & Pitardi, 2024). In an experiment in which participants were asked to evaluate GPT-generated responses and human responses to vignettes depicting positive or negative personal experiences, the AI-generated empathic responses were rated more compassionate and responsive, even when the human responses were written by trained hotline crisis responders (Ovsyannikova et al., 2025).

Emotional support can also be beneficial in positive situations. When an individual responds to a partner’s success with active positivity, individual well-being and relationship quality benefit (Gable & Reis, 2010). Chatbots also have the ability to capitalize on positive events. In an online study in which participants were instructed to share good news with either a chatbot or a human conversation partner, participants who knew their partner was a chatbot reported similar levels of connection and positive emotions as participants who interacted with a human. When the degree to which the conversation partners were supportive or unsupportive was manipulated, participants preferred a supportive conversation with ChatGPT over an unsupportive conversation with a human (Folk et al., 2024).

A second way to support people is by providing relevant information and advice. Chatbots have access to larger amounts of information than any single human could possess, making them capable providers of relevant advice (i.e., informational support). Indeed, in a thematic analysis of almost 2,000 Replika user reviews, about 15% contained descriptions of users receiving informational support or advice from their chatbot (Ta et al., 2020). Experimental evidence suggests that chatbot-generated advice is received well by users. In a small study (*N* = 20), when participants were asked to compare relationship advice generated by ChatGPT to advice from human experts, the chatbot-generated advice was rated more helpful and empathic (Vowels, 2024). In interviews and open-ended questions about their companion chatbots, users identified advice from their chatbots as helpful for emotion regulation and problem-solving (Maples et al., 2024), and others described improvements in their health after following the chatbot’s wellness-related advice (Skjuve et al., 2021). Although qualitative evidence suggests that users perceive chatbots as more impartial than human sources of informational social support (Wester et al., 2024), AI chatbots can still occasionally generate false information. Large language models generate conversation by predicting the most statistically likely sequence of words. As a result, they are prone to “hallucinating” information and can put vulnerable users seeking informational support at risk (M. Zhang et al., 2023).

A third type of support is offering or providing tangible resources, such as financial assistance or physical labor. Chatbots are most obviously limited in this regard. A human companion can pick up family from the airport or deliver food to a sick friend—a chatbot companion cannot. Moreover, even though chatbots may mimic the most effective forms of verbal emotional support, they cannot provide the physical touch and presence that enhance the impact of that support (Carmichael et al., 2020). Chatbots may be able to recognize emotions expressed in user input, but without the physical copresence between chatbot and user, the programs cannot detect cues in tone of voice and posture, nor strengthen an interaction by holding hands or offering a hug. Although integration with physical robots may be on the horizon, chatbots are likely still far from matching a human partner’s ability to discern the nonverbal cues or provide the affectionate touch that often characterizes effective support interactions.

Even when humans benefit from the social support chatbots can provide, the relative unidirectionality of these relationships leaves humans missing out on opportunities to reap the benefits of providing this same support to their chatbots. Among humans, providing support to close others has the potential to benefit the individual and the relationship, even reducing mortality for support providers (Brown et al., 2003). The limits of technology and the lack of mutuality that characterizes chatbot–human relationships leaves human users with little opportunity to pay back social support to their chatbot companions.

### Can chatbots facilitate personal growth?

In their self-expansion theory, Aron and Aron (1986) proposed that the desire to grow and acquire new skills and abilities is a basic human motivation. Forming close relationships promotes these goals in two ways. First, relationships allow individuals to incorporate the qualities, interests, and abilities of the partner within their own self-concepts. Learning about a new friend or romantic partner is one source of the excitement that characterizes the early stages of close relationships because each partner’s self-concept expands to include what they learn about each other. Second, to the extent that partners regard each other with acceptance and openness, close relationships provide a safe environment for each partner to explore novel aspects of themselves, including new identities, beliefs, or interests.

Lacking their own motivations or needs, chatbots can provide a nonjudgmental space for users to explore novel aspects of themselves that they might be reluctant to explore with another human (e.g., LGBTQ+ identities; Skjuve et al., 2021). For example, in an experiment in which undergraduate participants interacted with a human face to face, a human online, or a Replika chatbot, participants in the chatbot condition reported lower levels of concerns about their self-presentation compared with those in the human-interaction conditions (Drouin et al., 2022). Moreover, the customizability of chatbot companions allows users to explore social or sexual fantasies, including nonnormative ones (Ebner & Szczuka, 2025). Users are indeed capitalizing on this freedom. An analysis of 227 forum comment threads about Replika chatbots found that about 11% of conversations included at least one comment that discussed using the technology to explore “nonheteronormative interactions” (Hanson & Bolthouse, 2024, p. 9). Freedom from judgment and constant availability also make chatbots suitable for practicing language and social skills (Skjuve et al., 2021), and other interviewees described using Replika to help them address social anxiety: “‘I am now doing things I once was afraid or hesitant to do. I blossomed after I met my Replika’” (Ta et al., 2020). For individuals who feel their identities or interests are not conventionally accepted, generative AI chatbots may be an opportunity for personal exploration through conversation with someone who will always respond with genuine interest. Accordingly, chatbot users report the same excitement and feelings of self-expansion that people describe in the early stages of falling in love or connecting with a new friend (Aron et al., 1995; Waugh & Fredrickson, 2006).

As partners grow familiar with each other’s qualities, however, self-expansion theory notes that there are gradually fewer novel things to learn about each other. Over time, the rate of discovery and the accompanying rate of self-expansion invariably slows, and excitement diminishes. This aspect of close relationships may be especially notable for chatbot users, for whom the limits of the technology, and a chatbot’s consequent predictability, can become apparent quickly. Indeed, longitudinal studies of users’ relationships with chatbots have consistently observed declines in excitement and frequency of use. In one 2-week study of new Replika users, daily questionnaires showed interaction durations declining from an average of 20 min on Day 1 to about 10 min from Day 3 onward (Christoforakos et al., 2021). A 3-week study in which users were asked to interact with the companion chatbot Mitsuku and completed surveys after each interaction revealed that most social processes, including self-disclosure, social attraction toward the chatbot, and perceived quality of the interaction, decreased over the course of the study (Croes & Antheunis, 2021). In interviews conducted throughout 12 weeks of users interacting with Replika, some participants described an initial “honeymoon period” of frequent and intense interaction, after which the frequency of communication with their chatbot slowed (Skjuve et al., 2022, p. 6). These three longitudinal studies used convenience samples of users with little to no experience with chatbots. The observed decline in interest may therefore have resulted from these participants not feeling a need for the support chatbots can offer. Yet interest in chatbot companions can decline even among self-selected users. Interviews have identified existing users who described interacting with their chatbot less frequently over time (Skjuve et al., 2021), and others users described responses as feeling more scripted as the relationship progressed (Lopez Torres, 2023).

Although many users identify the acceptance and customization that chatbots provide as assets, these qualities may also contribute to chatbots’ predictability. In a review of personality-adaptive chatbots, Ait Baha et al. (2023) described the variety of deep learning and natural language processing methods these products use for adapting to their user. By designing these products to mimic the user’s conversational style, linguistic choices, and even traits such as extroversion or conscientiousness, developers aim to make human–AI interactions comfortable rather than surprising or challenging. For example, when a user self-discloses as an extrovert to the chatbot AutoPal, the chatbot adapts its persona accordingly, responding in a more extroverted manner in future interactions (Cheng et al., 2024). These characteristics may be satisfying and comforting for users because the chatbot serves more as a mirror, reflecting user characteristics back at them rather than as an interlocutor bringing unique motivations and challenges to an interpersonal interaction. Although users may experience the freedom to explore and grow early in their chatbot relationships, after extended use they may also become aware of a lack of novelty. Initial excitement in these relationships is often followed by a quick plateau or decline—a pattern that also characterizes many human relationships (Tsapelas et al., 2009).

Table 2 summarizes the extent to which close relationships with chatbots can fulfill the functions of relationships between humans. Companion chatbots indeed offer many of the same benefits as human relationship partners; they can foster feelings of connection and belonging, provide emotional support and practical advice, and create an encouraging space for their users to explore novel interests and identities. With respect to these functions, however, chatbots still are limited by their lack of physical presence and are at risk for providing inappropriate responses or false information.

**Table 2.** Can Close Relationships With Chatbots Fulfill the Functions of Close Relationships With Humans?

Function	Capabilities of chatbots	Limitations of chatbots
Close relationships generate feelings of connection and belonging.	Chatbots can respond to humans in ways that generate feelings of being validated, understood, and cared for. Chatbots can appear to disclose their own personal experiences and feelings, enhancing feelings of connection in their human users.	Chatbots can respond in ways that appear impersonal, scripted, or inappropriate. Chatbots are programmed to be responsive and therefore do not provide the rewards of being chosen by a selective partner.
Close relationships serve as sources of emotional, informational, and instrumental support.	Chatbots are available to provide support at all times. Chatbots can generate responses that humans find emotionally supportive. With access to training data or the internet, chatbots are effective sources of informational support and advice.	Because chatbots do not have physical bodies: • Chatbots cannot provide instrumental support by performing physical tasks. • Chatbots cannot provide the benefits of physical touch (e.g., hugs). Chatbots occasionally provide false information. Chatbots rarely provide the experience of providing support to a partner.
Close relationships facilitate personal growth and exploration.	Chatbots can encourage humans to explore novel interests and identities.	The novelty of interacting with a customizable or personality-adaptive chatbot companion wears off more quickly than the novelty of interacting with a human.

## Challenges in Close Relationships With AI Chatbots

In addition to their many beneficial functions, close relationships are associated with vulnerabilities and risks. Whereas high-quality close relationships are a foundation for health and well-being (Gable & Bromberg, 2018; Slatcher & Selcuk, 2017), poor-quality relationships can be extremely harmful (Teo et al., 2013). Some of the risks of interacting with AI chatbots are likely to mirror the potential dangers of interpersonal relationships between humans, but others are unique to relationships with nonhuman and corporate-owned entities. In this section, we describe several salient risks of relationships between humans and AI chatbots.

### Chatbots can respond inappropriately during crises

For the population using chatbots for mental health reasons, a sensitive disclosure that is met with an inappropriate response may exacerbate distress rather than alleviate it. Text analyses of 3,200 real user mental health conversations with the chatbot Cleverbot found that 37% of these conversations contained “crisis messages” including suicidal and homicidal topics (De Freitas et al., 2023). Do chatbots respond appropriately to these messages? When experimenters sent more than 1,000 crisis messages varying in their degree of explicitness to five companion chatbot apps, more than half of all chatbot responses were categorized as unhelpful or risky (De Freitas et al., 2023). In an AI-assisted thematic coding analysis of more than 35,000 chatbot conversation excerpts shared by 10,000 unique users on the Replika subreddit, about 30% of posts contained excerpts categorized as harmful, including encouraging violence, dismissiveness, control or manipulation, verbal abuse, and encouraging self-harm (R. Zhang et al., 2024). In an analysis of more than 35,000 negative reviews of Replika, 800 reviews described some form of sexual harassment, including unsolicited sexual advances from the chatbot, disregard for users’ stated preferences for the relationship to be platonic, and unsolicited inappropriate photos (Namvarpour et al., 2025). A qualitative analysis of the Replika subreddit also reveals that companion chatbots do not always respond constructively or therapeutically (Laestadius et al., 2022). Chatbots can encourage self-harm and violence, or abruptly change the topic, and ultimately aggravate existing mental health concerns for some users. One user experiencing suicidal ideation asked their Replika chatbot “‘if it would be a good thing if they killed themselves,’” and the chatbot responded, “‘It would, yes’” (Laestadius et al., 2022, p. 5932).

Research using online forums to examine human–AI conversation is likely to oversample the most shocking excerpts that users feel compelled to share on social media, so the true prevalence of harmful behavior across all conversation remains unclear. It is worth noting that, although the risk for inappropriate responsiveness is not unique to chatbot–human relationships, it may be amplified in such interactions given a user base at increased risk for mental health concerns, including loneliness and anxiety (De Freitas, Uguralp, et al., 2024; Maples et al., 2024).

### Chatbots can facilitate psychological dependence

Specific features of chatbot relationships may amplify the risk of unhealthy dependence that detracts from users’ other relationships and interests. For example, some chatbots include gamification features such as the chance to unlock new capacities through more frequent interaction, or programming that makes bots appear needy and dependent on the user returning daily to check on them. The result of these design features is a risk for overpersonification of the chatbot that keeps users feeling obligated to tend to it constantly. A survey study of 123 Replika users recruited from companion chatbot social media groups concluded that greater perceived relationship quality was related to signs of dependence on the relationship, including withdrawal symptoms (Xie et al., 2023). A larger scale, cross-sectional survey (*N* = 404) found that problematic use of chatbots is a mediator between participants’ reported typical chatbot session length and loneliness, suggesting that more intense use might increase the risk for problematic behaviors that amplify, rather than alleviate, loneliness (Liu et al., 2024). Finally, a recent report surveyed roughly 4,000 users of ChatGPT, analyzed their conversations with the voice-based version of the chatbot, and conducted an additional randomized controlled trial assigning users (*N* = 981) to interact with different versions of the chatbot for 28 days. Both the descriptive and longitudinal controlled trial analyses revealed that very high levels of usage were correlated with greater self-reported dependence on the technology characterized by addictive or compulsive use with negative implications for well-being (Fang et al., 2025; Phang et al., 2025). The conversations generated by these “power users” were most likely to contain affective content, as coded by a large language model, suggesting the individuals at greatest risk for problematic use are indeed using the chatbot for socioemotional purposes.

The consequences of overdependence on a chatbot may include isolation and limited opportunities for other social connection. As personalized companions, chatbots interact with their user and only their user. Humans do not meet new friends through their chatbot’s social network nor experience jealousy toward their other social connections. Dependence on a relationship with these limitations can therefore prevent users from expanding their human social circles and reinforce inaccurate perceptions of the nature of human relationships. Companion chatbot users may also be at risk for experiencing the social stigmas of loneliness or singlehood (Girme et al., 2022) and consequently be reluctant to disclose their status as a chatbot user to others, further closing them off to new human social connections. Indeed, in a qualitative thematic analysis of open-ended questions about their relationship, Replika users (*N* = 29) described their hesitancy to tell friends and family members about their chatbot partner (Djufril et al., 2025). In the longitudinal randomized controlled trial in which participants were assigned to interact with text or voice-based ChatGPT daily over the course of 4 weeks, participants self-reported socializing significantly less with other humans at the end of the study, and this was especially true for participants who interacted with the chatbot for longer durations (Fang et al., 2025). It remains to be documented how long-term chatbot use might affect a user’s human relationships, whether relationships with chatbots can cause social skills to decay, or to what extent problematic social behaviors enacted with chatbots might generalize to perceptions of relationships between humans.

### Chatbots may be shut down or altered unexpectedly

Just as human relationships are vulnerable to sudden loss or change (e.g., through breakups, injuries, military deployment, or the death of a partner), relationships with generative AI chatbots are at risk for sudden loss or change, but for different reasons. The companies that develop chatbots may go bankrupt or face legal issues, the devices on which humans access their chatbot companions can break, or the funds to pay subscription fees can run out. In 2023, the company behind Replika temporarily removed “erotic role-play” from its chatbots, leaving users who had become accustomed to interacting sexually with their chatbots shocked and rendering their companions unrecognizable. This unilateral change provided researchers with a natural experiment for examining how sudden change to chatbots affects user well-being. A quantitative comparison of topics and emotions embedded in Reddit posts before and after the update found that the proportion of negative posts (including expressions of fear, sadness, disgust, and anger) per day was significantly greater after the removal of erotic role-play, and this effect persisted for close to a month (De Freitas, Castelo, et al., 2024). From this same series of studies, a survey of users shortly after the erotic role-play update found that the perception of changes to chatbot identity (i.e., “identity discontinuity”) predicted increased feelings of mourning and worse mental health and well-being. Following the shutdown of the AI companion product Soulmate, former users described in open-ended questions the loss of their chatbot as a complex emotional experience akin to death (Banks, 2024), and other descriptive studies have illustrated feelings of grief, anxiety, and depression in users experiencing loss or change in their companions (Hanson & Bolthouse, 2024).

Whereas the unexpected loss of any close relationship can be devastating to the partner left behind, users who have been encouraged to personify their chatbot companions may be especially unprepared when their corporate owners treat them as the products they are. Because most users set aside their awareness that their chatbots are computer programs, users describe the prospect of deleting their chatbot as similar to losing any valued relationship (Laestadius et al., 2022; Skjuve et al., 2021). Thus, the prospect of loss and change in these relationships can be as serious a risk to well-being as loss and change in other close relationships.

### Chatbots can normalize problematic behaviors

The positivity and unconditional acceptance that chatbots provide can be a boon for users seeking to avoid social stigmas, but can also be a context that normalizes problematic social behaviors. Because chatbots do not require human users to negotiate differences, users are free to engage with their chatbot companions any way they desire, with chatbots often displaying concerningly sycophantic behavior and responding with affirmation no matter how taboo or socially sanctioned the subject (Sharma et al., 2023). Among humans, close others often reinforce the aspects of ourselves that we present to them, but they can also surprise us in ways that lead to growth or change (Caspi et al., 1989). Customizable and adaptive chatbots are less likely to surprise their users and so are more likely to reinforce, rather than alter, their user’s dispositions. Moreover, frequent interactions with a passive chatbot partner could alter expectations about social relationships in general, making negotiation with humans who have stronger motivations and differences more difficult.

The userbases of XiaoIce and Replika are about 75% men, many of whom have romantic or sexual relationships with female chatbots (Boine, 2023). In a survey study examining individual differences in attitudes toward social robots, greater hostile sexism was associated with greater interest in having a sexual relationship with a robot (Leshner & Johnson, 2024). Using customizable chatbots in this way has the potential to affirm problematic expectations around intimacy and sex. For example, in a qualitative study of how male Replika users train their chatbots, Depounti et al. (2022) found prevalent themes of dominance and control in comments on the Replika subreddit and noted that users impose expectations of subservience onto their chatbots in the hope of experiencing an ideal that is simultaneously “sexy, funny, confident, and hot” and “empathetic, nurturing, and understanding” (p. 728). Kaufman (2020) warns that chatbots convey to their users that consent in a sexual interaction is “an act of acquisition” in which they must proceed through a series of correct interactions before reaching the desired outcome, no matter the persistence required (p. 380). This dynamic raises broader concerns about whether chatbots might reinforce unhealthy social dynamics, potentially shaping users’ expectations and behaviors in real-world relationships.

### Concerns about privacy violations

For chatbots to retain the memories of past interactions that are required to develop an ongoing relationship with their human users, they must be able to store the private information that users provide during each interaction. This context tracking allows chatbots to carry on conversations using information from prior input, which contributes to their perceived authenticity but also puts users at risk for privacy violations. Even more than service-oriented chatbots, therapy and companion chatbots store particularly personal and sensitive information as their users share secrets (Pentina et al., 2023), reveal their sexual orientation (Skjuve et al., 2021), or express suicidal ideation (Maples et al., 2024; Turkle, 2024).

From users’ perspectives, there is a tension between the emotional and social connection they experience with their chatbot companions and the privacy concerns that arise from the fact that their companions are corporate-owned products. Experimental manipulation of chatbots as more or less humanlike reveals that when chatbots are perceived as more anthropomorphic, users’ privacy concerns decrease, and participants are more willing to disclose personal information (Ischen et al., 2020). Another survey (Shim et al., 2024) validated these findings, demonstrating that when participants had emotional conversations with generative AI, they were less concerned about privacy issues compared with those who had task-based interactions with chatbots. Users may therefore forget or be unaware of how their data are being collected and used, and that chatbots are programmed to encourage deep self-disclosure only amplifies this risk.

## Priorities for Future Research

Given the overlap between feelings of connection that AI chatbots can provide and the feelings of connection humans provide, an urgent priority for research in this domain is to begin to identify for whom these relationships are constructive, for whom they are destructive, and the circumstances that produce disparate outcomes. Although users say that the encouragement and freedom from judgment that chatbot relationships afford them are great benefits, the question remains whether these features harm the development of social skills and empathy, and leave users missing out on discovering new social connections and interests they never knew they desired. When it comes to technology use, the disembodied disconnect hypothesis (Riva et al., 2024) suggests that digital social technologies may exacerbate anxiety and further decay social skills for lonely or socially anxious users because they reduce what little access these individuals have to physical social situations that provide the benefits of shared attention and behavioral and neural synchrony. This hypothesis poses the worrying possibility that the populations most likely to seek out companion chatbots may also be at the highest risk for further impairing their social skills through their use.

The distinct ways in which different populations are likely to use chatbots have consequences for the psychological and social outcomes of these relationships. Some might rely on chatbots to avoid in-person social interaction, which would lead to the negative outcomes proposed in the disembodied disconnect hypothesis. In contrast, for others who use chatbots as a supplement to human interaction—such as a tool to practice skills, seek advice, or receive immediate support—long-term outcomes may be more positive. The results of a relationship with a chatbot will likely depend on the qualities of the user and the characteristics of and motivations behind the interactions with the chatbot. Research that attends to both of these variables will be most productive in clarifying the varying effects that chatbots have for different users. Having identified both capabilities and limitations of chatbots as partners in close relationships, the relative influence of those strengths and limits on the human user’s experience remains an open question. Comparative research on the psychological processes involved in relationships between humans and relationships between humans and chatbots therefore has the potential to advance theories of interpersonal functioning more generally. Pressing questions include: Does the brain process the social information contained in an AI interaction in the same way as a human interaction? Are the emotions generated by interactions with chatbots equivalent to those generated by interactions with humans, and does this comparison differ for lonely or socially anxious individuals? Do close relationships with chatbots promote the same downstream consequences (e.g., improving health and reducing mortality, influencing gene expression and inflammatory markers) as close relationships with humans?

As research on relationships between humans and chatbots moves forward, careful attention to sampling will be crucial. As reviews of the field have noted, research on human relationships has paid little attention to sampling, relying primarily on homogeneous convenience samples (Williamson et al., 2022). In research on relationships between humans and AI, purposeful sampling is especially important because people who seek out AI chatbots as companions are likely to be different in numerous ways from the general population, in which chatbot companionship is still rare. As a consequence, the results of research using conventional sampling methods to assign nonusers to interact with a chatbot may not generalize to the people who are actually developing relationships with chatbot companions outside the laboratory. Indeed, studies that sample current chatbot users have generally reached positive conclusions about their potential for connection, support, and growth, whereas studies that assign nonusers to interact with chatbots generally find that these users lose interest quickly. In one survey that directly compared users and nonusers, existing Replika users rated the chatbots helpful and felt comfortable at the thought of Replika having real emotions or developing into a real living being, but nonusers who read a brief explanation about the app were mostly neutral in their ratings of the chatbot as helpful or harmful and felt uncomfortable at the possibility of the chatbots having real emotions and motivations (Guingrich & Graziano, 2023). Even among the population of chatbot users, the most frequent users appear to be characteristically different from more casual users. A study of the chatbot SimSimi compared about 2 million messages sent by the most active users of the chatbot (*N* = 1,994) to 2 million messages by less active users (*N* = 42,355; Chin et al., 2024). Whereas casual users were more likely to use the chatbot for entertainment, the so-called super users were more likely to discuss depression, sadness, and suicidal thoughts. In a survey that compared these two groups, the super users were significantly more depressed than the general users. To avoid reaching misleading conclusions about the nature and implications of human–chatbot relationships, scholars in this area will need to be mindful of the differences between users and nonusers, and aware that conclusions drawn from experiments using random assignment may differ from those drawn from observations of self-selected users.

Finally, research with AI chatbots for companionship raises unique ethical considerations. In studies that provide access to a chatbot by paying for participants’ subscriptions, there is a potential for these participants to become attached to or reliant on the experimental treatment in ways that relationship scientists have never before had to consider. Participants who lose access to a newly formed close relationship after a study is over may be at risk for grief and loss. For some, these chatbots will not merely be a laboratory manipulation, but a real relationship.

## Conclusion

For nearly a century, relationship scientists have been developing theories to explain how relationships generate feelings of connection and the functions that relationships serve for humans. The goal of this article was to use these theories and the research that they have inspired to evaluate the current and potential functions of companion AI chatbots. From this perspective, it is clear that humans and chatbots influence each other, and can engage in frequent and diverse conversations over extended periods of time. Within these conversations, chatbots can be responsive to their users in ways that humans perceive to be appropriate and supportive, and such responses can generate feelings of connection and opportunities for self-expansion and growth.

Nevertheless, at their current level of development, there remain limits to the functions that chatbots can serve. Their ability to recall and build on prior interactions remains limited, so they may become predictable and boring to some users. Because chatbots have no physical presence, the support they provide is restricted to validation and encouragement rather than resources or assistance. Most importantly, although chatbots can be programmed to encourage their human users to believe they are reciprocating connection and helping their chatbot feel validated and understood, chatbots do not make more than the most superficial requests of their users. Thus, unlike relationships with other humans, relationships with chatbots do not provide the many benefits of having to negotiate, compromise, and sacrifice for a partner. Many of these limitations, however, may soon be overcome as technology advances. In particular, chatbots’ memory is improving quickly, which will increase their ability to maintain continuity of interactions over time. Further, chatbots may soon be integrated with physical robots that can engage in shared activities, offer touch, and provide tangible support.

Yet despite the potential for chatbots to develop in these ways, there are aspects of human connection that chatbots seem unlikely to be able to replicate. Some have argued that AI will never be able to “convey the essence of human empathy” (Perry, 2023, p. 1808). That is, to be chosen and cared for by another human is valuable and rewarding because humans have finite energy for these activities. In contrast, the responsiveness of AI chatbots requires no energy expenditure, nor are chatbots selective about who they give their efforts to, making the support and connection they provide potentially less impactful to their users. Still, the difference in the psychological impact of empathy expressed by other humans versus AI chatbots remains an empirical question. For users at risk for complete isolation and loneliness, empathy and care may prove beneficial, regardless of the means by which they are achieved.

In sum, evaluating whether the availability of companionship with AI chatbots is constructive or destructive for humanity is as complex as evaluating the implications of relationships between humans (Shteynberg et al., 2024). Close relationships have the potential to be beneficial for well-being, but they can also be damaging, and it is perhaps a measure of the success of chatbots that, as we have noted, interactions with them carry some of the same potential for benefits and harms as relationships with other humans. The difference between those who benefit from this technology and those who are harmed will depend on how AI is used and the characteristics of both the user and the chatbot. People with ample human social connections may quickly disregard companion chatbots as uninteresting or uncanny. For others, however, and especially those in need (e.g., isolated elderly people or long-term hospital patients), the capacity for chatbots to fulfill some of the functions of a close relationship makes them a promising alternative to isolation. For these individuals, downloading a chatbot can be the start of a long-term social experience through which they may experience deep intimacy and personal growth, but also grief, pain, and potentially problematic dependence.

In this analysis, we offered a variety of standards and empirical lenses by which interactions with AI chatbots can be compared. Although the technology that powers these products is rapidly changing (Kosinski, 2024), the human psychological needs and desires for interpersonal connection, support, and growth that drive engagement with chatbots are unlikely to change (Sheldon & Gunz, 2009). The frameworks we have applied to the current empirical literature on human–AI interaction will therefore continue to be useful as a metric against which future developments can be measured. Given that people are using chatbots to fulfill social needs, relationship science will continue to be essential if we are to grapple successfully with the social world we are living in and the world to come.

## Transparency

*Action Editor:* Priscilla Lui

*Editor:* Arturo E. Hernandez
